# Supplementary material for: Epigenome-wide association analysis of infant bronchiolitis severity: a multicenter prospective cohort study
Source: Nat Commun. 2023 Sep 7;14:5495. doi: 10.1038/s41467-023-41300-y (PMC10485022; doi:10.1038/s41467-023-41300-y)
Supplement: Supplementary file 1 — Supplementary Information [file 41467_2023_41300_MOESM1_ESM.pdf]

Online Supplement

Epigenome-wide association analysis of infant bronchiolitis severity: A multicenter prospective cohort study

Zhaozhong Zhu, ScD; Yijun Li, PhD; Robert J. Freishtat, MD, MPH; Juan C. Celedón, MD, DrPH; Janice A. Espinola, MPH; Brennan Harmon, MA; Andrea Hahn, MD, MS; Carlos A. Camargo, Jr., MD, DrPH; Liming Liang, PhD*; and Kohei Hasegawa, MD, MPH, PhD*

* These authors jointly supervised this work

Corresponding Author: Dr. Zhaozhong Zhu, Department of Emergency Medicine, Massachusetts General Hospital, 125 Nashua Street, Suite 920, Boston, MA 02114. (E-mail: zzhu5@mgh.harvard.edu; Tel: 617-726-3925. Fax: 617-724-4050)

Table of Contents

Supplementary Methods 3

Study Design, Setting, and Participants 3

Data Collection.................... 3

DNA Extraction 4

Blood DNA Methylation Profiling and Quality Control 4

Blood Immune Cell-Type Deconvolution and Differentially Methylated Cell Types.................... 6

Project Viva DNA Methylation Dataset 7

Genetics of DNA Methylation Consortium (GoDMC) Methylation Quantitative Trait Loci (meQTL) Dataset 7

UK Biobank Asthma Genome-Wide Association Study (GWAS) Dataset.................... 8

UK Biobank Lung Function GWAS Dataset 8

Mendelian randomization Analysis 9

Supplementary Table 1. Principal investigators at the 17 participating sites in MARC-35....................11

Supplementary Table 2. Comparisons between analytic and non-analytic cohorts in infants with bronchiolitis....................12

Supplementary Table 3. Baseline characteristics and clinical course of 575 infants hospitalized for bronchiolitis by asthma status at age six years 14

Supplementary Table 4. Twenty-two severity-related differential methylated regions in infant hospitalized with bronchiolitis 16

Supplementary Table 5. Association of seven blood cell types and bronchiolitis severity 17

Supplementary Figure 1. Sample selection and quality control flow diagram 18

Supplementary Figure 2. DNA methylation probe-level quality control flow diagram 19

Supplementary Figure 3. Distribution of 794,177 CpGs by chromosome and profiling platform 20

Supplementary Figure 4. RSV or RV infection-stratified analysis for the association of the 33 DMCs with the bronchiolitis severity 21

Supplementary References 22

Supplementary Methods

Study Design, Setting, and Participants

We analyzed data from a multicenter prospective cohort study of infants hospitalized for bronchiolitis—the 35th Multicenter Airway Research Collaboration (MARC-35) study [1]. MARC-35 is coordinated by the Emergency Medicine Network (EMNet), an international research collaboration with 247 participating hospitals. At 17 sites across 14 U.S. states (**Table E1**), MARC-35 enrolled infants (aged <1 year) who were hospitalized with an attending physician diagnosis of bronchiolitis during three bronchiolitis seasons (November 1 to April 30) from 2011 to 2014. The diagnosis of bronchiolitis was made according to the American Academy of Pediatrics bronchiolitis guidelines, defined as acute respiratory illness with a combination of rhinitis, cough, tachypnoea, wheezing, crackles, or retraction.[2] We excluded infants with a preexisting heart and lung disease, immunodeficiency, immunosuppression, or gestational age of <32 weeks, history of previous bronchiolitis hospitalization, or those who were transferred to a participating hospital >24 hours after initial hospitalization. All patients were treated at the discretion of the treating physicians. The institutional review board at each participating hospital approved the study with a written informed consent obtained from the parent or guardian.

Of 1,016 infants enrolled in the MARC-35 cohort, the current analysis investigated 625 infants hospitalized for bronchiolitis who were selected for high-quality blood DNA methylation testing (**Figures 1** and **E1**).

Data Collection

Clinical data (patients' demographic characteristics, and family, environmental, and medical history, and details of the acute illness) were collected via structured interview and chart reviews [1]. All

data were reviewed at the EMNet Coordinating Centre (Boston, MA), and site investigators were queried about missing data and discrepancies identified by manual data checks.

In addition to the clinical data, whole blood specimens were collected by trained site investigators using the standardized protocol that was utilized in a previous cohort study of children with bronchiolitis [1, 3]. All sites used the same collection equipment and collected the samples within 24 hours of hospitalization. The whole blood specimens were immediately placed on ice and then stored at −80°C.

DNA Extraction

Frozen blood pellet tubes were quickly thawed in a 37°C water bath for 5-10 min. The thawed blood pellets were then transferred to 2mL Lysing Matrix M tubes (MP Biomedicals, Irvine, CA) and processed in the FastPrep-24™ 5G bead beating grinder (MP Biomedicals, Irvine, CA) for 2 cycles at a speed of 6.0 m/s for 40 sec. The homogenized samples were then spun at 1,000xg for 30 sec in a benchtop centrifuge to remove any remaining blood clot particles. The supernatant was then collected (leaving behind the blood clot particles) and transferred to a new tube. The supernatant was then treated as whole blood and was processed for DNA isolation using the Puregene Blood Kit (Qiagen, Germantown, MD) by following the DNA Purification from Whole Blood protocol in the kit handbook. The resultant DNA pellets were resuspended in 50 µL of DNA Hydration Solution (Qiagen, Germantown, MD) and rehydrated by incubating at 65°C for 1 hr and overnight at room temperature.

Blood DNA Methylation Profiling and Quality Control

Peripheral blood samples were collected from 630 infants who were eligible for the current analysis. For each sample, DNA methylation was measured at 863,904 cytosine-phosphate-guanine

(CpG) sites using the Illumina Infinium MethylationEPIC BeadChip (Illumina, San Diego, CA) in the Mass General Brigham Personalized Medicine Biobank Genomics Core Lab. To ensure the quality of the DNA methylation data, we followed the existing data preprocessing pipeline in the R/Bioconductor package *minfi* [4]. We applied multiple sample-level and probe-level quality control filters (**Figures E1-E2**). Samples that met any of the following criteria were removed: (a) showed poor bisulfite conversion, extension, hybridization, staining, or specificity as indicated by the built-in control probes; (b) had low overall array intensity, defined as having a median log2 Meth or Unmeth signal less than 10; (c) had more than 1% of probes with a detection p-value greater than 0.01; (d) showed a discrepancy between predicted sex, obtained based on methylation data using the getSex() function in *minfi*, and reported sex in the clinical records. In addition, we utilized the information of 59 single nucleotide polymorphisms (SNPs) measured by the built-in SNP probes on the EPIC array and compared this information with previously measured genotyping microarray data in the same sample. Samples with mismatched SNP information between the EPIC array and the genotyping microarray were excluded. A flowchart describing the sample-level quality control can be found in the **Figure E1**. Then, we applied probe-level quality control filters to remove poorly measured CpG sites. These filters included (a) removing probes with a detection p-value greater than 0.01 in more than 1% of all samples, (b) removing cross-reactive probes that co-hybridize to multiple locations in the genome [5], (c) removing probes that overlap genetic variants at target CpGs or at single-base extension [5], and (d) removing probes with genetic variants (minor allele frequency >5%) within 10 bases from the body of probes [5]. The cross-reactive probes and probes overlapping genetic variants have been identified in a previous study [5]. Following the quality control steps, we applied the single-sample normal-exponential normalization using the out-of-band probes (ssNoob) procedure to conduct background correction and dye bias correction [6]. **Figure E2** provides detailed information regarding the probe-level quality control. A total of 625 infant blood

samples with DNA methylation data of 794,177 CpG sites passed the quality control and were included to the downstream analysis.

Blood Immune Cell-Type Deconvolution and Differentially Methylated Cell Types

We used EpiDISH R package [7] to infer the blood cell type composition. EpiDISH provides function to infer the fractions of a priori known cell subtypes present in a sample representing a mixture of such cell-types. We used Robust Partial Correlations method [8] to infer seven blood cell-types, including B cells, cytotoxic T (T_C) cells, helper T (T_H) cells, eosinophils, monocytes, neutrophils, and natural killer (NK) cells, from our EWAS data. After estimating cell-type fractions, we then investigated whether the differentially methylated CpGs (DMCs) are differentially methylated (i.e., hypermethylation or hypomethylation) in these cell types using CellDMC function from EpiDISH R package [7]. We corrected multiple testing using the Benjamini-Hochberg false discovery rate (FDR) method [9].

Human Early Life Exposome (HELIX) Project cis-Expression Quantitative Trait Methylation (eQTM) Dataset

Detailed information on the quality control procedures used in the publicly available HELIX Project eQTM data was described in a previous study [10]. The HELIX Project has collected blood specimens from 823 European ancestry children (mean age, 8 years). For the cis-eQTM data, blood DNA methylation and gene expression were measured with the Illumina 450K and the Affymetrix HTA v2 arrays, respectively. To test associations between DNA methylation levels and gene expression levels in cis (cis-eQTMs), each gene was paired to CpGs closer than ±500kb from its transcription start site (TSS), either upstream or downstream. For each gene, the TSS was defined based on HTA-2.0

annotation, using the start position for transcripts in the + strand, and the end position for transcripts in the - strand. CpGs position was obtained from Illumina 450K array annotation. Only CpGs in autosomal chromosomes (from chromosomes 1–22) were tested. The relationship between methylation levels and expression of nearby genes (±500kb window centered at the TSS) was assessed by fitting 13.6 million linear regressions adjusting for age, sex, cohort, and blood cell composition [10].

Project Viva DNA Methylation Dataset

Detailed information on the imputation and quality control procedures used in the publicly available Project Viva DNA Methylation data was described in a previous study [11]. The Project Viva is a prospective pre-birth cohort study. This study collected nasal swabs from the anterior nares of 547 children (mean-age 12.9 years) and measured DNA methylation with the Infinium MethylationEPIC BeadChip (850K) [11]. We retrieved the publicly available summary statistics from the nasal EWAS of ten respiratory and immune related traits, including asthma, allergic asthma, bronchodilator response, forced expiratory volume in one second (FEV1), forced vital capacity (FVC), FEV1/FVC, allergic rhinitis, total immunoglobulin E (IgE), specific IgE, and fractional exhaled nitric oxide (FeNO). We extracted the summary statistics of these ten traits for 29 (i.e., only 29 out of 33 available in Project Viva) differentially methylated CpGs (DMCs) for bronchiolitis severity from the current study and compared their effect size and significance level.

Genetics of DNA Methylation Consortium (GoDMC) Methylation Quantitative Trait Loci (meQTL) Dataset

Detailed information on the imputation and quality control procedures used in the publicly available GoDMC meQTL data was described in a previous study [12]. The GoDMC standardized meta-

analysis and data integration across 36 population-based and disease datasets. There are ~10 million genotypes imputed to the 1000 Genomes Project reference panel [13] and quantified DNAm at 420,509 sites using Illumina HumanMethylation BeadChips (450K) in whole blood derived from up to 27,750 European participants [12]. We have used both local (cis-meQTL) and distal (trans-meQTL) data. We removed the SNPs with minor allele frequency of <1%. We also restricted our analysis to the bi-allelic SNPs.

UK Biobank Asthma Genome-Wide Association Study (GWAS) Dataset

Detailed information on the imputation and quality control procedures used in the UK Biobank asthma data was described in our previous studies [14, 15, 16, 17]. In brief, first, we identified all UK Biobank participants with asthma by using the data fields 6152 (self-reported physician-diagnosis of asthma), 20002 (non-cancer illness disease code), 41202 (ICD-10-CM primary diagnosis in the hospital), and 41204 (ICD-10-CM secondary diagnosis in the hospital). The ICD-10-CM diagnosis codes of J45 were used for the identification of asthma. Second, we performed a GWAS analysis with adjusting for age, sex, genotyping array, and 30 ancestry principal components using BOLT-LMM v2.3 [18]. The Haplotype Reference Consortium panel data [19] were used as the reference panel for imputation. This reference panel has a larger number of haplotypes than the 1000G reference panel; therefore, it is expected to produce a higher imputation performance [19]. We removed the SNPs with Hardy-Weinberg equilibrium of $<1\times10^{-12}$, minor allele frequency of <1%, and imputation quality score of <0.8. We also restricted our analysis to the bi-allelic SNPs. All genetic analysis was restricted to subjects of European ancestry.

UK Biobank Lung Function GWAS Dataset

Detailed information on the imputation and quality control procedures used in the UK Biobank lung function data was described in our previous study. In brief, Pre-bronchodilator FEV_1 and FVC were

measured by trained health technicians following recommended procedure using Vitalograph Pneumotrac 6800 spirometer [20]. The Vitalograph Pneumotrac 6800 spirometer was chosen because it is extensively in observational studies and clinical trials, and fulfilled various key requirements (e.g. conformed to ATS requirements, validated, reliable, robust, easy to use, IT data download). Up to three measurements of lung function within a maximum of 6 minutes (since more attempts over a more prolonged period were not considered acceptable for participants) were conducted. FEV1, FVC and FEV1/FVC were adjusted for age, age^2, sex, height, smoking status (ever vs. never) and assessment center in a linear regression model [21]. The resulting residuals were inverse normal transformed [21]. The residuals were used as the outcome for the GWAS analysis. Second, we performed a GWAS analysis testing the association between the genotype and lung function traits' residuals using BOLT-LMM v2.3 [18]. The Haplotype Reference Consortium panel [19] were used as the reference panel for imputation. We removed the SNPs with Hardy-Weinberg equilibrium of $<1\times10^{-12}$, minor allele frequency of <1%, and imputation quality score of <0.8. We also restricted our analysis to the bi-allelic SNPs. All genetic analysis was restricted to subjects of European ancestry.

Mendelian randomization Analysis

We conducted Mendelian randomization analysis using TwoSampleMR [22] R package under default settings to investigate putative causal relationships between meQTL from GoDMC [12] and four respiratory traits from UK Biobank, including asthma [14, 15, 16, 17], FEV1 [23], FVC [23], FEV1/FVC [23]. There was no overlapping subjects between the exposures and outcomes in the Mendelian randomization analysis. All subjects in the Mendelian randomization analysis are European ancestry. To ensure the robustness of Mendelian randomization analysis, we have constructed the instrumental variables based on exposures containing a minimum of 10 linkage disequilibrium-independent SNPs with P-value less

than 5×10^{-8}. As a result, a total of four CpGs (cg09541576, cg12547959, cg12896170, and cg15848159) with high quality meQTL instrumental variables were included for the Mendelian randomization analysis. Prior to running Mendelian randomization, we harmonized the effect of SNP for both meQTL and respiratory traits and removed SNPs with strand-ambiguity. To comprehensively evaluate the causal effect between exposures and outcome, we applied three Mendelian randomization approaches, inverse variance-weighted method [24], MR–Egger regression method [25], a weighted median method [26]. We corrected multiple testing using the Benjamini-Hochberg FDR method [9] in the Mendelian randomization analysis.

Supplementary Table 1. Principal investigators at the 17 participating sites in MARC-35

Amy D. Thompson, MD	Alfred I. duPont Hospital for Children, Wilmington, DE
Federico R. Laham, MD, MS	Arnold Palmer Hospital for Children, Orlando, FL
Jonathan M. Mansbach, MD, MPH	Boston Children's Hospital, Boston, MA
Vincent J. Wang, MD, MHA and Susan Wu, MD	Children's Hospital of Los Angeles, Los Angeles, CA
Michelle B. Dunn, MD and Jonathan M. Spergel, MD, PhD	Children's Hospital of Philadelphia, Philadelphia, PA
Juan C. Celedón, MD, DrPH	Children's Hospital of Pittsburgh, Pittsburgh, PA
Michael R. Gomez, MD, MS-HCA and Nancy Inhofe, MD	The Children's Hospital at St. Francis, Tulsa, OK
Brian M. Pate, MD and Henry T. Puls, MD	The Children's Mercy Hospital & Clinics, Kansas City, MO
Stephen J. Teach, MD, MPH	Children's National Medical Center, Washington, D.C.
Richard T. Strait, MD and Stephen C. Porter, MD, MSc, MPH	Cincinnati Children's Hospital and Medical Center, Cincinnati, OH
Ilana Y. Waynik, MD	Connecticut Children's Medical Center, Hartford, CT
Sujit Iyer, MD	Dell Children's Medical Center of Central Texas, Austin, TX
Michelle D. Stevenson, MD, MS	Norton Children's Hospital, Louisville, KY
Margaret Samuels-Kalow, MD, MPhil, Wayne G. Shreffler, MD, PhD and Ari R. Cohen, MD	Massachusetts General Hospital, Boston, MA
Anne K. Beasley, MD and Cindy S. Bauer, MD	Phoenix Children's Hospital, Phoenix, AZ
Thida Ong, MD and Markus Boos, MD, PhD	Seattle Children's Hospital, Seattle, WA
Charles G. Macias, MD, MPH	Texas Children's Hospital, Houston, TX

Supplementary Table 2. Comparisons between analytic and non-analytic cohorts in infants with bronchiolitis

Characteristics	Analytic cohort (n=625)	Non-analytic cohort (n=391)	P-value
**Demographics**			
Age (month), median (IQR)	3 (2–6)	3 (1–6)	0.01
Female sex	240 (38)	166 (43)	0.22
Race/ethnicity			0.03
Non-Hispanic White	287 (46)	143 (37)	
Non-Hispanic Black	136 (22)	103 (26)	
Hispanic	180 (29)	128 (33)	
Other or unknown	22 (4)	17 (4)	
Prematurity (32–36.9 weeks)	107 (17)	79 (20)	0.25
Birth weight (kg), median (IQR)	3.28 (2.90–3.60)	3.26 (2.90–3.54)	0.65
Mode of birth (cesarean delivery)	210 (34)	138 (36)	0.69
Previous breathing problems before the index hospitalization*			0.26
0	488 (78)	322 (82)	
1	106 (17)	54 (14)	
2	31 (5)	15 (4)	
Previous ICU admission	8 (1)	9 (2)	0.33
History of eczema	102 (16)	47 (12)	0.07
Lifetime antibiotic use	201 (32)	117 (30)	0.50
Ever attended daycare	153 (25)	81 (21)	0.19
Cigarette smoke exposure at home	101 (16)	55 (14)	0.42
Maternal smoking during pregnancy	101 (17)	46 (12)	0.05
Parental history of asthma	204 (33)	141 (36)	0.29
Parental history of eczema	122 (20)	76 (19)	1.00
**Clinical presentation**			
Weight at presentation (kg), median (IQR)	6.20 (4.90–7.92)	5.80 (4.40–7.44)	0.002
Respiratory rate at presentation (per minute), median (IQR)	48 (40–60)	50 (40–60)	0.20
Oxygen saturation at presentation			0.77
<90%	56 (9)	35 (9)	
90–93%	99 (16)	56 (15)	

≥94%	455 (75)	294 (76)	
Blood eosinophilia (≥4%)	60 (11)	36 (11)	0.93
IgE sensitization (%)	128 (21)	76 (19)	0.75
Length of hospitalization (day), median (IQR)	2 (1–3)	2 (1–4)	0.009
Corticosteroid use during hospitalization	82 (13)	50 (13)	0.95
**Respiratory virus**			
RSV infection	473 (76)	348 (89)	<0.001
RV infection	133 (21)	81 (21)	0.89
**Acute clinical outcome**			
Positive pressure ventilation use[†]	30 (5)	25 (6)	0.34

Abbreviations: ICU, intensive care unit; IgE, immunoglobulin E; IQR, interquartile range; RSV, respiratory syncytial virus; RV, rhinovirus.

Data are no. (%) of infants unless otherwise indicated. Percentages may not equal 100, because of rounding and missingness. Chi-square tests were used to compare proportions for categorical variables (such as sex), and Mann–Whitney U tests to compare median for continuous variables (such as age).

[*] Defined as an infant having a cough that wakes him or her at night or causes emesis, or when the child has wheezing or shortness of breath without cough.

[†] Defined as the use of continuous positive airway pressure ventilation and/or mechanical ventilation during the hospitalization.

Supplementary Table 3. Baseline characteristics and clinical course of 575 infants hospitalized for bronchiolitis by asthma status at age six years

Characteristics	PPV* use (n=30)	Non-PPV use (n=595)	P-value
**Demographics**			
Age (month), median (IQR)	2 (1–4)	3 (2–6)	0.04
Female sex	13 (43)	227 (38)	0.71
Race/ethnicity			0.71
Non-Hispanic white	15 (50)	272 (46)	
Non-Hispanic black	4 (13)	132 (22)	
Hispanic	10 (33)	170 (29)	
Other or unknown	1 (3)	21 (4)	
Prematurity (32–36.9 weeks)	6 (20)	101 (17)	0.86
Birth weight (kg), median (IQR)	3.28 (2.78-3.63)	3.28 (2.90–3.60)	0.67
Mode of birth (cesarean delivery)	12 (41)	198 (34)	0.54
Previous breathing problems before the index hospitalization†			0.91
0	24 (80)	464 (78)	
1	5 (17)	101 (17)	
2	1 (3)	30 (5)	
Previous ICU admission	1 (3)	7 (1)	0.85
History of eczema	3 (10)	99 (17)	0.48
Lifetime antibiotic use	8 (27)	193 (32)	0.65
Ever attended daycare	5 (17)	148 (25)	0.42
Cigarette smoke exposure at home	2 (7)	99 (17)	0.23
Maternal smoking during pregnancy	5 (17)	96 (17)	1.00
Parental history of asthma	6 (20)	198 (33)	0.19
Parental history of eczema	8 (27)	114 (19)	0.44
**Clinical presentation**			
Weight at presentation (kg), median (IQR)	5.45 (4.25-6.96)	6.20 (4.90–8.00)	0.02
Respiratory rate at presentation (per minute), median (IQR)	48 (38-64)	48 (40–60)	0.62
Oxygen saturation at presentation			<0.001
<90%	9 (32)	47 (8)	

90–93%	7 (25)	92 (16)	
≥94%	12 (43)	443 (76)	
Blood eosinophilia (≥4%)	5 (19)	55 (11)	0.34
IgE sensitization (%)	9 (30)	119 (20)	0.28
Hospital length-of-stay (day), median (IQR)	9 (6-12)	2 (1-3)	<0.001
Corticosteroid use during hospitalization	14 (47)	68 (11)	<0.001
**Respiratory virus**			
RSV infection	21 (70)	452 (76)	0.60
RV infection	3 (10)	130 (22)	0.19

Abbreviations: ICU, intensive care unit; IgE, immunoglobulin E; IQR, interquartile range; PPV, positive pressure ventilation; RSV, respiratory syncytial virus; RV, rhinovirus. Chi-square tests were used to compare proportions for categorical variables (such as sex), and Mann–Whitney U tests to compare median for continuous variables (such as age).

Data are no. (%) of infants unless otherwise indicated. Percentages may not equal 100, because of rounding and missingness.
* Defined as the use of continuous positive airway pressure ventilation and/or mechanical ventilation during the hospitalization.
† Defined as an infant having a cough that wakes him or her at night or causes emesis, or when the child has wheezing or shortness of breath without cough.

Supplementary Table 4. Twenty-two severity-related differential methylated regions in infant hospitalized with bronchiolitis

Region_chr	start	end	no.CpGs	z_sidak_p	Transcript	GeneSymbol	distance2TSS	Promoter
chr6	3849235	3849818	21	7.56×10^{-13}	uc003mvu.3	*FAM50B*	0	TRUE
chr5	14326044	14326531	4	9.05×10^{-12}	uc003jfh.1	*TRIO*	32923	FALSE
chr5	43037123	43037666	7	6.73×10^{-9}	uc003jnf.3	*ANXA2R*	2781	FALSE
chr2	27665079	27665306	6	2.93×10^{-5}	uc002rks.3	*KRTCAP3*	0	TRUE
chr6	142623433	142623767	5	3.68×10^{-5}	uc003qix.2	*ADGRG6*	377	FALSE
chr1	15271952	15272383	8	5.04×10^{-5}	uc001avp.2	*KAZN*	15656	FALSE
chr6	33282736	33282896	8	1.96×10^{-4}	uc003oeb.3	*ZBTB22*	2616	FALSE
chr8	77913262	77913341	5	6.87×10^{-4}	uc022awe.1	*PEX2*	0	TRUE
chr19	11784647	11785062	8	1.88×10^{-4}	uc021upi.1	*ZNF833P*	0	TRUE
chr10	45360781	45360969	3	6.15×10^{-4}	uc001jbk.1	*TMEM72-AS1*	94168	FALSE
chr5	132113601	132113860	9	1.17×10^{-3}	uc003kxt.2	*SEPTIN8*	-40	TRUE
chr17	40838861	40839022	3	3.26×10^{-3}	uc002iay.3	*CNTNAP1*	4229	FALSE
chr10	93058376	93058636	3	2.25×10^{-3}	uc010qnl.2	*HECTD2-AS1*	312581	FALSE
chr11	1036679	1036865	5	3.33×10^{-3}	uc001lsw.2	*MUC6*	0	TRUE
chr3	112013130	112013231	3	6.44×10^{-3}	uc003dyu.3	*SLC9C1*	-56	TRUE
chr1	203734256	203734559	6	3.00×10^{-3}	uc001haa.3	*LAX1*	0	TRUE
chr4	185369108	185369164	3	1.69×10^{-2}	uc003iwf.4	*IRF2*	26562	FALSE
chr19	2588376	2588629	3	4.47×10^{-3}	uc002lwd.2	*GNG7*	114117	FALSE
chr7	154684327	154684562	4	6.09×10^{-3}	uc003wlm.3	*DPP6*	681980	FALSE
chr3	122296369	122296613	8	6.77×10^{-3}	uc003efm.2	*PARP15*	0	TRUE
chr2	220108094	220108407	6	1.05×10^{-2}	uc010zkx.2	*GLB1L*	0	TRUE
chr3	10805960	10806243	6	2.30×10^{-2}	uc003bvx.1	*LINC00606*	-83	TRUE

Abbreviations: chr, chromosome; CpG, cytosine-phosphate-guanine; TSS, transcription-start site.

Supplementary Table 5. Association of seven blood cell types and bronchiolitis severity

Cell type	Effect Estimate	SE	P	FDR
B cells	-0.02	0.01	0.12	0.14
NK cells	-0.01	0.01	0.01	0.01
Helper T cells	-0.06	0.02	0.00	0.01
Cytotoxic T cells	-0.02	0.01	0.06	0.08
Monocytes	-0.02	0.01	0.01	0.01
Neutrophils	0.13	0.03	0.00	7.80×10^{-5}
Eosinophils	-0.01	0.00	0.14	0.14

Abbreviations: FDR, false discovery rate; SE, standard error.

Logistic regression tests were used to investigate the association between cell types and bronchiolitis severity. The analysis was adjusted for multiple comparisons using the Benjamini-Hochberg false discovery rate (FDR) method.

Supplementary Figure 1. Sample selection and quality control flow diagram

This figure shows the overall sample selection and quality control process. The differences in the analytic and non-analytic cohorts are summarized in **Supplementary Table 2**.

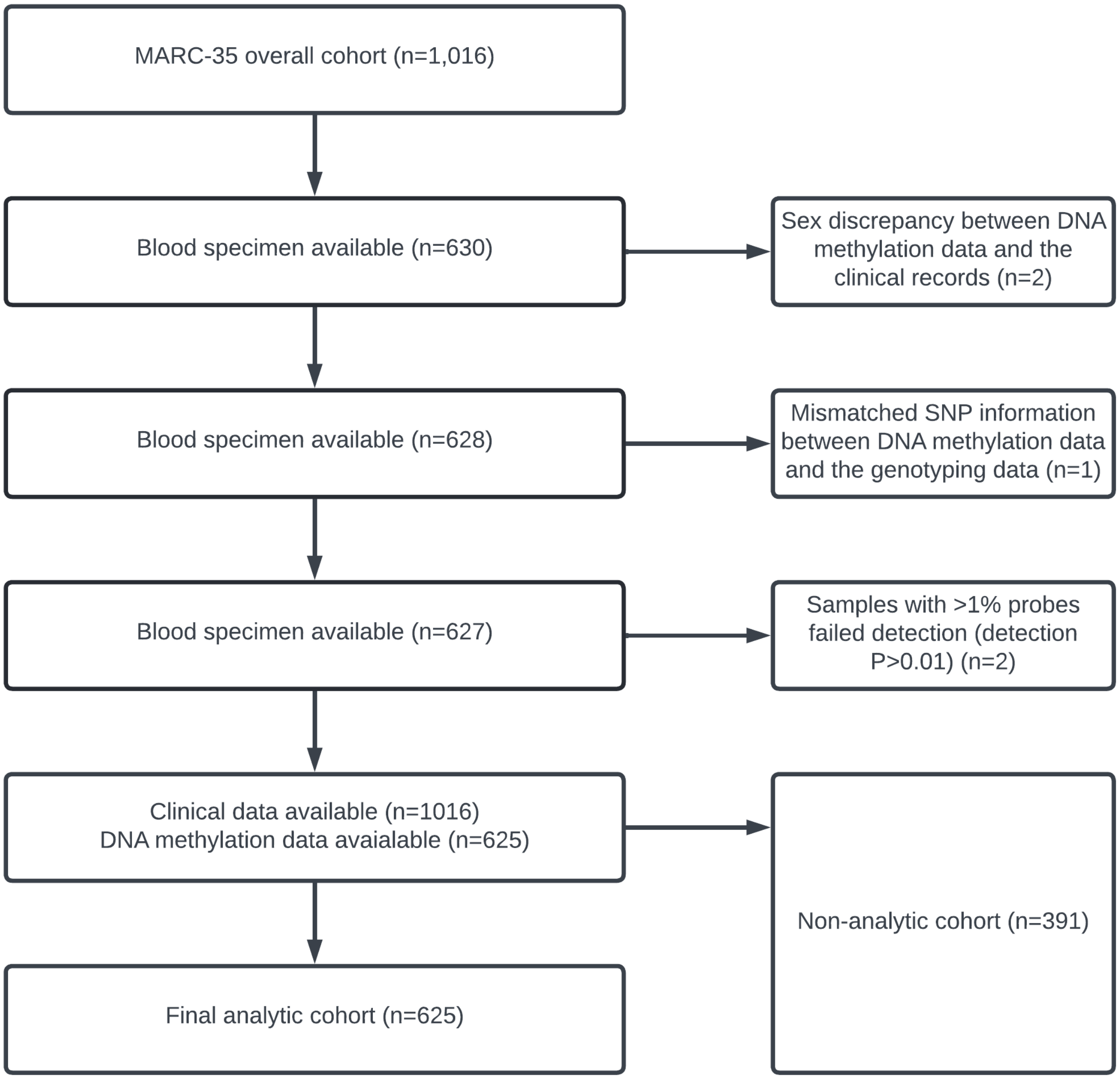

Supplementary Figure 2. DNA methylation probe-level quality control flow diagram

This figure shows the DNA methylation probe-level quality control process.

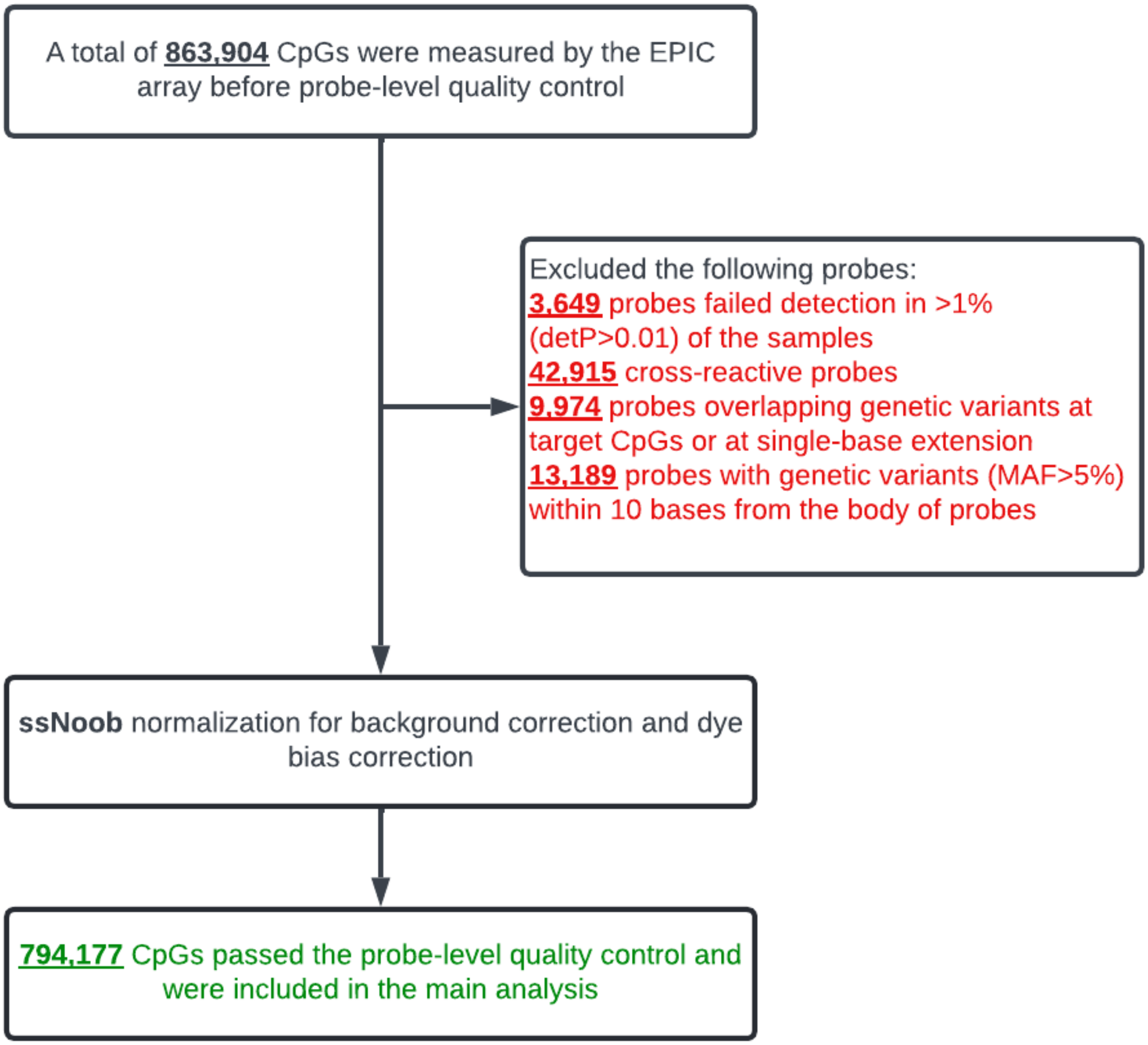

Supplementary Figure 3. Distribution of 794,177 CpGs by chromosome and profiling platform

This figure shows the distribution of 794,177 CpGs after the quality control process by chromosome and profiling platform. Pink color denotes 27K platform, green color denotes 450K platform, blue color denotes EPIC platform.

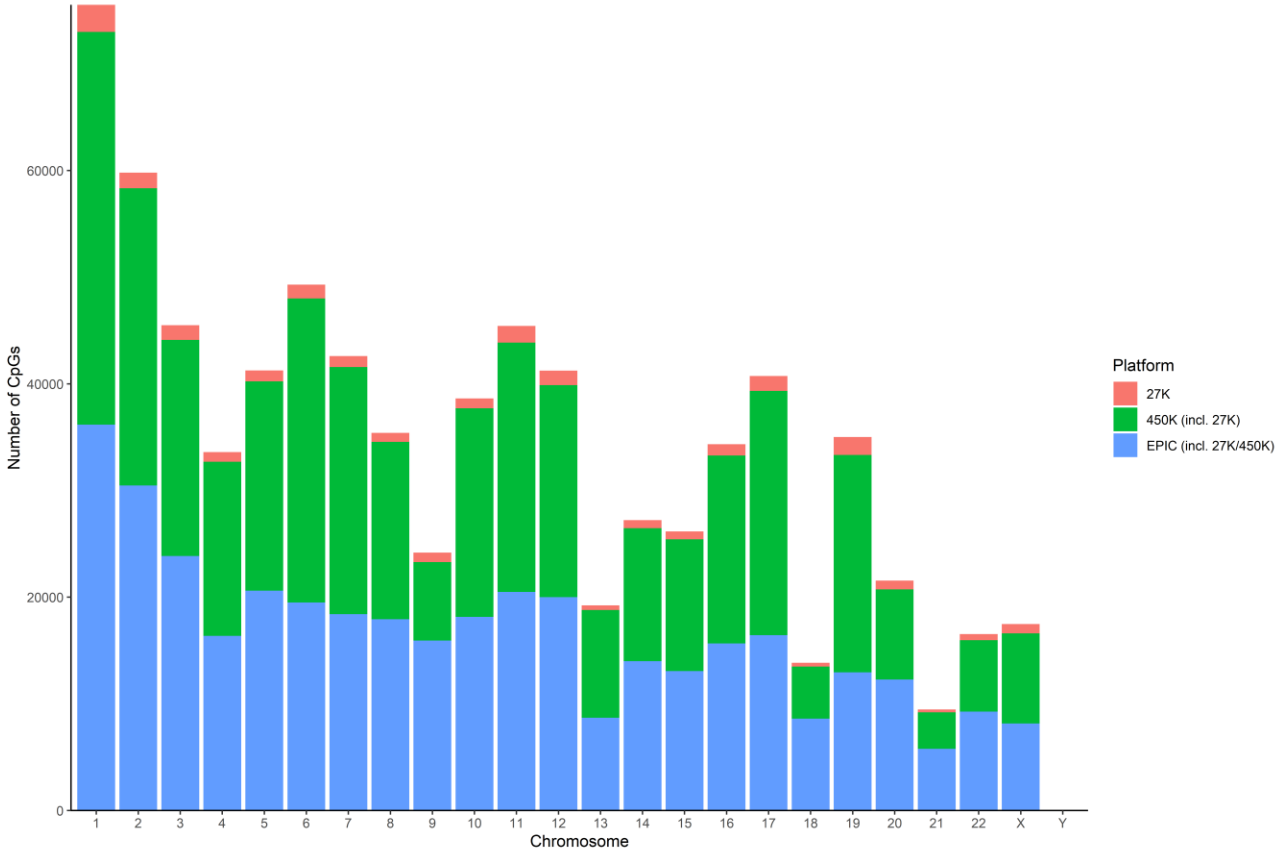

Supplementary Figure 4. RSV or RV infection-stratified analysis for the association of the 33 DMCs with the bronchiolitis severity

A) RSV infection-stratified analysis for the association of the 33 DMCs with the risk of PPV use.
B) RV infection-stratified analysis for the association of the 33 DMCs with the risk of PPV use.
Abbreviations: CpG, cytosine-phosphate-guanine; DMC: differentially methylated CpGs; FDR, false discovery rate; RSV, respiratory syncytial virus; RV, rhinovirus.

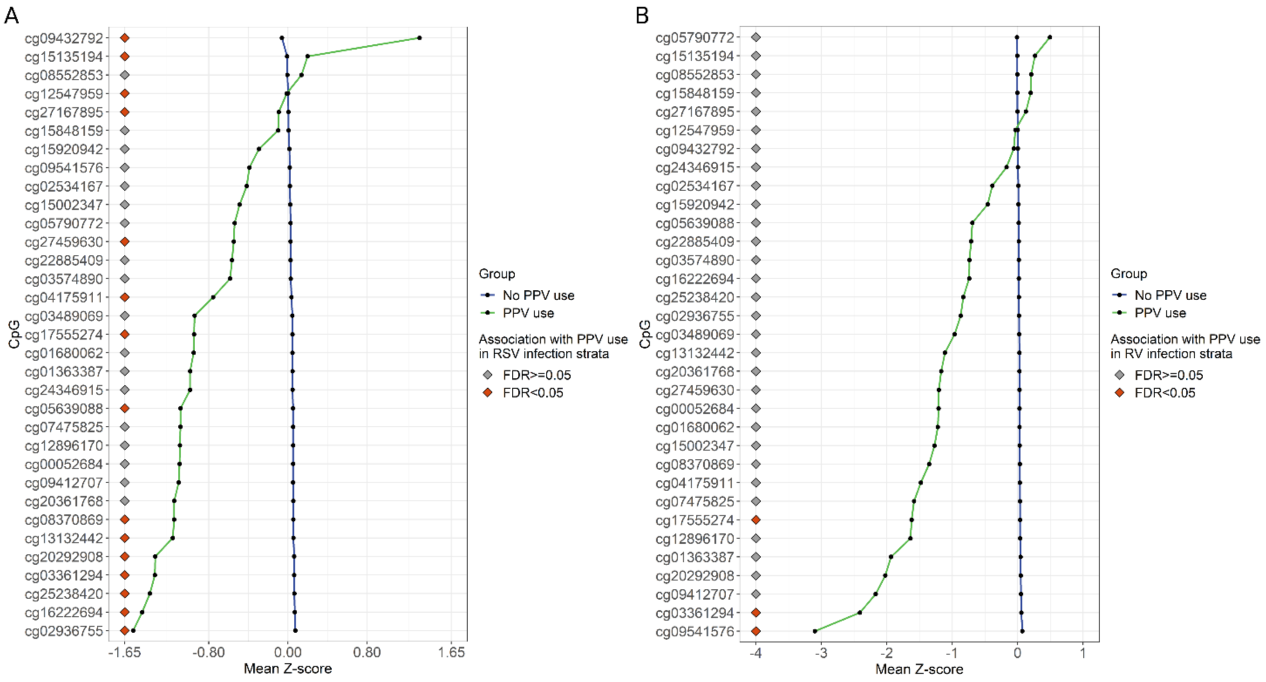

Supplementary References

1. Hasegawa K*, et al.* Association of nasopharyngeal microbiota profiles with bronchiolitis severity in infants hospitalised for bronchiolitis. *Eur Respir J* **48**, 1329-1339 (2016).
2. Ralston SL*, et al.* Clinical practice guideline: the diagnosis, management, and prevention of bronchiolitis. *Pediatrics* **134**, e1474-1502 (2014).
3. Hasegawa K*, et al.* Respiratory syncytial virus genomic load and disease severity among children hospitalized with bronchiolitis: multicenter cohort studies in the United States and Finland. *J Infect Dis* **211**, 1550-1559 (2015).
4. Aryee MJ*, et al.* Minfi: a flexible and comprehensive Bioconductor package for the analysis of Infinium DNA methylation microarrays. *Bioinformatics* **30**, 1363-1369 (2014).
5. Pidsley R*, et al.* Critical evaluation of the Illumina MethylationEPIC BeadChip microarray for whole-genome DNA methylation profiling. *Genome Biol* **17**, 208 (2016).
6. Fortin JP, Triche TJ, Jr., Hansen KD. Preprocessing, normalization and integration of the Illumina HumanMethylationEPIC array with minfi. *Bioinformatics* **33**, 558-560 (2017).
7. Zheng SC, Breeze CE, Beck S, Teschendorff AE. Identification of differentially methylated cell types in epigenome-wide association studies. *Nat Methods* **15**, 1059-1066 (2018).
8. Newman AM*, et al.* Robust enumeration of cell subsets from tissue expression profiles. *Nat Methods* **12**, 453-457 (2015).
9. Benjamini Y, Hochberg Y. Controlling the False Discovery Rate: A Practical and Powerful Approach to Multiple Testing. *Journal of the Royal Statistical Society Series B (Methodological)* **57**, 289-300 (1995).
10. Ruiz-Arenas C*, et al.* Identification of autosomal cis expression quantitative trait methylation (cis eQTMs) in children's blood. *Elife* **11**, (2022).
11. Cardenas A*, et al.* The nasal methylome as a biomarker of asthma and airway inflammation in children. *Nat Commun* **10**, 3095 (2019).
12. Min JL*, et al.* Genomic and phenotypic insights from an atlas of genetic effects on DNA methylation. *Nat Genet* **53**, 1311-1321 (2021).
13. Genomes Project C*, et al.* An integrated map of genetic variation from 1,092 human genomes. *Nature* **491**, 56-65 (2012).
14. Zhu Z*, et al.* A genome-wide cross-trait analysis from UK Biobank highlights the shared genetic architecture of asthma and allergic diseases. *Nat Genet* **50**, 857-864 (2018).
15. Zhu Z*, et al.* Shared genetics of asthma and mental health disorders: a large-scale genome-wide cross-trait analysis. *Eur Respir J* **54**, (2019).
16. Zhu Z*, et al.* Shared genetic and experimental links between obesity-related traits and asthma subtypes in UK Biobank. *J Allergy Clin Immunol* **145**, 537-549 (2020).
17. Zhu Z, Hasegawa K, Ma B, Fujiogi M, Camargo CA, Jr., Liang L. Association of asthma and its genetic predisposition with the risk of severe COVID-19. *J Allergy Clin Immunol* **146**, 327-329 e324 (2020).
18. Loh PR*, et al.* Efficient Bayesian mixed-model analysis increases association power in large cohorts. *Nat Genet* **47**, 284-290 (2015).
19. McCarthy S*, et al.* A reference panel of 64,976 haplotypes for genotype imputation. *Nat Genet* **48**, 1279-1283 (2016).
20. Standardization of Spirometry, 1994 Update. American Thoracic Society. *Am J Respir Crit Care Med* **152**, 1107-1136 (1995).
21. Shrine N*, et al.* New genetic signals for lung function highlight pathways and chronic obstructive pulmonary disease associations across multiple ancestries. *Nat Genet* **51**, 481-493 (2019).

22. Hemani G*, et al.* The MR-Base platform supports systematic causal inference across the human phenome. *Elife* **7**, (2018).
23. Zhu Z*, et al.* A large-scale genome-wide association analysis of lung function in the Chinese population identifies novel loci and highlights shared genetic aetiology with obesity. *Eur Respir J* **58**, (2021).

24. Burgess S, Scott RA, Timpson NJ, Davey Smith G, Thompson SG, Consortium E-I. Using published data in Mendelian randomization: a blueprint for efficient identification of causal risk factors. *Eur J Epidemiol* **30**, 543-552 (2015).
25. Bowden J, Davey Smith G, Burgess S. Mendelian randomization with invalid instruments: effect estimation and bias detection through Egger regression. *Int J Epidemiol* **44**, 512-525 (2015).
26. Bowden J, Davey Smith G, Haycock PC, Burgess S. Consistent Estimation in Mendelian Randomization with Some Invalid Instruments Using a Weighted Median Estimator. *Genet Epidemiol* **40**, 304-314 (2016).
