# Supplementary material for: Epigenome-wide association analysis of infant bronchiolitis severity: a multicenter prospective cohort study
Source: Nat Commun. 2023 Sep 7;14:5495. doi: 10.1038/s41467-023-41300-y (PMC10485022; doi:10.1038/s41467-023-41300-y)
Supplement: Supplementary file 3 — Description of Additional Supplementary Files [file 41467_2023_41300_MOESM3_ESM.pdf]

Description of Additional Supplementary Files

File Name: Supplementary Data 1
Description: Abbreviations: CpG, cytosine-phosphate-guanine; chr, chromosome; FDR, false discovery rate; pos: position; SE, standard error; TC, transcript cluster. All results were from the HELIX project. No statistical test was conducted in the current study. The analysis from the HELIX project was adjusted for multiple comparisons using the Benjamini-Hochberg FDR method.
