# Supplementary material for: Epigenome-wide association analysis of infant bronchiolitis severity: a multicenter prospective cohort study
Source: Nat Commun. 2023 Sep 7;14:5495. doi: 10.1038/s41467-023-41300-y (PMC10485022; doi:10.1038/s41467-023-41300-y)
Supplement: Supplementary file 5 — Reporting Summary [file 41467_2023_41300_MOESM5_ESM.pdf]

nature portfolio

Corresponding author(s): Zhaozhong Zhu

Last updated by author(s): August 10, 2023

Reporting Summary

Nature Portfolio wishes to improve the reproducibility of the work that we publish. This form provides structure for consistency and transparency in reporting. For further information on Nature Portfolio policies, see our Editorial Policies and the Editorial Policy Checklist.

Statistics

For all statistical analyses, confirm that the following items are present in the figure legend, table legend, main text, or Methods section.

n/a	Confirmed	
☐	☒	The exact sample size (*n*) for each experimental group/condition, given as a discrete number and unit of measurement
☐	☒	A statement on whether measurements were taken from distinct samples or whether the same sample was measured repeatedly
☒	☐	The statistical test(s) used AND whether they are one- or two-sided<br>*Only common tests should be described solely by name; describe more complex techniques in the Methods section.*
☐	☒	A description of all covariates tested
☐	☒	A description of any assumptions or corrections, such as tests of normality and adjustment for multiple comparisons
☐	☒	A full description of the statistical parameters including central tendency (e.g. means) or other basic estimates (e.g. regression coefficient) AND variation (e.g. standard deviation) or associated estimates of uncertainty (e.g. confidence intervals)
☐	☒	For null hypothesis testing, the test statistic (e.g. *F*, *t*, *r*) with confidence intervals, effect sizes, degrees of freedom and *P* value noted<br>*Give P values as exact values whenever suitable.*
☒	☐	For Bayesian analysis, information on the choice of priors and Markov chain Monte Carlo settings
☐	☒	For hierarchical and complex designs, identification of the appropriate level for tests and full reporting of outcomes
☐	☒	Estimates of effect sizes (e.g. Cohen's *d*, Pearson's *r*), indicating how they were calculated

Our web collection on statistics for biologists contains articles on many of the points above.

Software and code

Policy information about availability of computer code

Data collection	R package "minfi" v1.44.0 was used for raw DNA methylation data processing and quality control.
Data analysis	R package "Meffil" v1.3.6 was used for EWAS analysis. Online software eFORGE 2.0 was used for DHS enrichment analysis. R package "EpiDISH" v2.14.1 was used to infer the blood cell type composition. R package "TwoSampleMR" v0.5.6 was used for Mendelian randomization analysis. BOLT-LMM v2.3 was used for UK Biobank GWAS analysis.

For manuscripts utilizing custom algorithms or software that are central to the research but not yet described in published literature, software must be made available to editors and reviewers. We strongly encourage code deposition in a community repository (e.g. GitHub). See the Nature Portfolio guidelines for submitting code & software for further information.

Data

Policy information about availability of data

All manuscripts must include a data availability statement. This statement should provide the following information, where applicable:

- Accession codes, unique identifiers, or web links for publicly available datasets
- A description of any restrictions on data availability
- For clinical datasets or third party data, please ensure that the statement adheres to our policy

Data availability: The EWAS summary statistics generated in this study are available at http://lianglab.rc.fas.harvard.edu/BronchiolitisSeverityEWAS/. In addition, the raw data that support the findings of this study will be available on the NIH/NIAID ImmPort under Accession ID: SDY2306 through controlled access to be compliant

with the informed consent forms of MARC-35 study and the genomic data sharing plan. All other data are publicly available through the original studies' website. Project Viva data are available at https://figshare.com/articles/dataset/The_Nasal_Methylome_as_a_Biomarker_of_Asthma_and_Airway_Inflammation_in_Children/8285612/1. GoDMC data are available at http://mqtldb.godmc.org.uk/. UK Biobank data are available at https://www.ebi.ac.uk/gwas/. GENCODE data are available at https://www.gencodegenes.org/. UCSC RefSeq data are available at https://genome.ucsc.edu/cgi-bin/hgTrackUi?g=refGene.

Research involving human participants, their data, or biological material

Policy information about studies with human participants or human data. See also policy information about sex, gender (identity/presentation), and sexual orientation and race, ethnicity and racism.

Reporting on sex and gender	Sex of each participants was used in this study. We adjusted sex as one of the confounders in the EWAS analysis.
Reporting on race, ethnicity, or other socially relevant groupings	Race/ethinicity of each participants was used in this study. We adjusted race/ethinicty as one of the confounders in the EWAS analysis.
Population characteristics	Please see Tables 1 and E2 for detailed subject characteristics.
Recruitment	At 17 medical centers across 14 U.S. states (Table E1), MARC-35 enrolled infants (age <1 year) who were hospitalized with an attending physician diagnosis of bronchiolitis during three bronchiolitis seasons in 2011-2014. The diagnosis of bronchiolitis was made according to the American Academy of Pediatrics bronchiolitis guidelines, defined as an acute respiratory illness with a combination of rhinitis, cough, tachypnea, wheezing, crackles, or retraction (50). We excluded infants with preexisting heart or lung disease, immunodeficiency, immunosuppression, or gestational age of <32 weeks. All infants were managed at the discretion of the treating physicians. Of 1,016 infants enrolled in the MARC-35 cohort, the current study investigated 625 infants with high-quality blood DNA methylation data (Figure E1).
Ethics oversight	The institutional review board at each participating hospital approved the study with written informed consent obtained from the parent or guardian. The following is a list of the participating hospitals:<br><br>Alfred I. duPont Hospital for Children, Wilmington, DE<br>Arnold Palmer Hospital for Children, Orlando, FL<br>Boston Children's Hospital, Boston, MA<br>Children's Hospital of Los Angeles, Los Angeles, CA<br>Children's Hospital of Philadelphia, Philadelphia, PA<br>Children's Hospital of Pittsburgh, Pittsburgh, PA<br>The Children's Hospital at St. Francis, Tulsa, OK<br>The Children's Mercy Hospital & Clinics, Kansas City, MO<br>Children's National Medical Center, Washington, D.C.<br>Cincinnati Children's Hospital and Medical Center, Cincinnati, OH<br>Connecticut Children's Medical Center, Hartford, CT<br>Dell Children's Medical Center of Central Texas, Austin, TX<br>Norton Children's Hospital, Louisville, KY<br>Massachusetts General Hospital, Boston, MA<br>Phoenix Children's Hospital, Phoenix, AZ<br>Seattle Children's Hospital, Seattle, WA<br>Texas Children's Hospital, Houston, TX

Note that full information on the approval of the study protocol must also be provided in the manuscript.

Field-specific reporting

Please select the one below that is the best fit for your research. If you are not sure, read the appropriate sections before making your selection.

☒ Life sciences ☐ Behavioural & social sciences ☐ Ecological, evolutionary & environmental sciences

For a reference copy of the document with all sections, see nature.com/documents/nr-reporting-summary-flat.pdf

Life sciences study design

All studies must disclose on these points even when the disclosure is negative.

Sample size	Of 1,016 infants enrolled in the MARC-35 cohort, the current analysis investigated 625 infants hospitalized for bronchiolitis who were selected for high-quality blood DNA methylation testing (Figures 1 and E1). The blood DNA methylation testing was based on the availability of blood specimens in the cohort.
Data exclusions	We excluded 5 samples and 69,727 CpG probes after DNA methylation quality control process. Please see Tables E1 and E2 for more details.
Replication	In the current study, we have identified 46 differentially methylated CpGs for bronchiolitis severity outcome. While nearly half of the identified CpGs were associated with related respiratory and immune traits in an independent and publicly available Project Viva EWAS study, our inferences warrant external replication using the same bronchiolitis severity outcome. However, to our best knowledge, DNA methylation

	data with the same outcome is not currently available.
Randomization	Sample plates and chips during DNA methylation profiling were fully randomized to ensure to minimize potential confounding by batch effects.
Blinding	No blinding was applied in this study since MARC-35 is a prospective (a type of observational) cohort study.

Reporting for specific materials, systems and methods

We require information from authors about some types of materials, experimental systems and methods used in many studies. Here, indicate whether each material, system or method listed is relevant to your study. If you are not sure if a list item applies to your research, read the appropriate section before selecting a response.

Materials & experimental systems

n/a	Involved in the study
☒	☐ Antibodies
☒	☐ Eukaryotic cell lines
☒	☐ Palaeontology and archaeology
☒	☐ Animals and other organisms
☐	☒ Clinical data
☒	☐ Dual use research of concern
☒	☐ Plants

Methods

n/a	Involved in the study
☒	☐ ChIP-seq
☒	☐ Flow cytometry
☒	☐ MRI-based neuroimaging

Clinical data

Policy information about clinical studies
All manuscripts should comply with the ICMJE guidelines for publication of clinical research and a completed CONSORT checklist must be included with all submissions.

Clinical trial registration	NA
Study protocol	Clinical data (study participants' demographic characteristics, family, environmental, medical history, and details of the acute illness) were collected via structured interview and chart reviews using a standardized protocol.
Data collection	Clinical data (study participants' demographic characteristics, family, environmental, medical history, and details of the acute illness) were collected via structured interview and chart reviews using a standardized protocol. After the index hospitalization for bronchiolitis, trained interviewers began interviewing parents/legal guardians by telephone at 6-month intervals in addition to medical record review by physicians. All data were reviewed at the Emergency Medicine Network Coordinating Center at Massachusetts General Hospital (Boston, MA, USA). Whole blood specimens were collected within 24 hours of hospitalization using a standardized protocol. The details of the data collection and measurement methods are described in the Supplementary Methods.
Outcomes	The outcome of interest was higher disease severity defined by the use of positive pressure ventilation (PPV) (i.e., continuous positive airway pressure and/or intubation with mechanical ventilation) during the hospitalization for bronchiolitis.
